# MicroRNA-195-5p Inhibits Intracerebral Hemorrhage-Induced Inflammatory Response and Neuron Cell Apoptosis

**DOI:** 10.3390/ijms251910321

**Published:** 2024-09-25

**Authors:** Yi-Cheng Tsai, Chih-Hui Chang, Yoon Bin Chong, Chieh-Hsin Wu, Hung-Pei Tsai, Tian-Lu Cheng, Chih-Lung Lin

**Affiliations:** 1 Graduate Institute of Medicine, College of Medicine, Kaohsiung Medical University, Kaohsiung 807, Taiwan; iiidns11@hotmail.com (Y.-C.T.); chchang20@gmail.com (C.-H.C.); bin99068@hotmail.com (Y.B.C.); tlcheng@kmu.edu.tw (T.-L.C.); 2 Division of Neurosurgery, Department of Surgery, Kaohsiung Medical University Hospital, Kaohsiung 807, Taiwan; wujoeys@gmail.com (C.-H.W.); carbugino@gmail.com (H.-P.T.); 3 Department of Surgery, School of Medicine, College of Medicine, Kaohsiung Medical University, Kaohsiung 807, Taiwan; 4 Regenerative Medicine and Cell Therapy Research Center, Kaohsiung Medical University, Kaohsiung 807, Taiwan; 5 Department of Biochemistry, School of Post Baccalaureate Medicine, College of Medicine, Kaohsiung Medical University, Kaohsiung 807, Taiwan; 6 Drug Development and Value Creation Research Center, Kaohsiung Medical University, Kaohsiung 807, Taiwan; 7 Department of Biomedical Science and Environmental Biology, Kaohsiung Medical University, Kaohsiung 807, Taiwan

**Keywords:** brain hemorrhage, microRNA, neurobehavior

## Abstract

Intracerebral hemorrhage (ICH) is a severe condition characterized by bleeding within brain tissue. Primary brain injury in ICH results from a mechanical insult caused by blood accumulation, whereas secondary injury involves inflammation, oxidative stress, and disruption of brain physiology. miR-195-5p may participate in ICH pathology by regulating cell proliferation, oxidative stress, and inflammation. Therefore, we assessed the performance of miR-195-5p in alleviating ICH-induced secondary brain injury. ICH was established in male Sprague–Dawley rats (7 weeks old, 200–250 g) via the stereotaxic intrastriatal injection of type IV bacterial collagenase, after which miR-195-5p was administered intravenously. Neurological function was assessed using corner turn and forelimb grip strength tests. Protein expression was assessed by western blotting and ELISA. The miR-195-5p treatment significantly improved neurological function; modulated macrophage polarization by promoting anti-inflammatory marker (CD206 and Arg1) production and inhibiting pro-inflammatory marker (CD68 and iNOS) production; enhanced Akt signalling, reduced oxidative stress by increasing Sirt1 and Nrf2 levels, and attenuated inflammation by decreasing NF-κB activation; inhibited apoptosis via increased Bcl-2 and decreased cleaved caspase-3 levels; and regulated synaptic plasticity by modulating NMDAR2A, NMDAR2B, BDNF, and TrkB expression and ERK and CREB phosphorylation. In conclusion, miR-195-5p exerts neuroprotective effects in ICH by reducing inflammation and oxidative stress, inhibiting apoptosis, and restoring synaptic plasticity, ultimately restoring behavioral recovery, and represents a promising therapeutic agent that warrants clinical studies.

## 1. Introduction

Intracerebral hemorrhage (ICH) is a medical condition characterized by bleeding of the brain tissue resulting from various factors, including hypertension, cerebral amyloid angiopathy, and underlying structural lesions [1,2,3]. ICH accounts for 15–20% of all strokes [4,5] and is associated with higher mortality and disability rates than ischemic strokes, making it an important global health concern [6,7]. ICH is a type of catastrophic stroke characterized by high mortality and morbidity rates [8]. A rapid response to ICH is crucial, as approximately half of the patients succumb within the first 48 h, and less than a fifth regain full neural functionality [9]. The high morbidity and mortality rates associated with ICH are associated with both primary and secondary brain injuries [10]. Primary injury is primarily caused by a mechanical insult to the brain tissue due to the rapid accumulation of blood within the parenchyma [11]. Secondary injury occurs due to various factors, including the release of blood metabolites, inflammation, oxidative stress, and the disruption of normal physiological processes [10,12]. ICH triggers a robust inflammatory response, characterized by the invasion of blood components across the compromised blood–brain barrier (BBB), leading to vasogenic edema, BBB breakdown, and glial cell death [13]. Apoptosis, a form of programmed cell death, has also been implicated in neuronal loss surrounding hematomas [14]. Given the lack of evidence-based surgical treatment strategies for ICH, secondary brain injury has become an important focus of clinical research because the underlying mechanisms may affect treatment outcomes and represent promising intervention targets [15].

MicroRNAs (miRNAs) are small non-coding RNA molecules that play crucial roles in post-transcriptional gene regulation. These molecules, typically composed of 20–22 nucleotides, modulate gene expression by binding to specific mRNA molecules and inhibiting their translation into proteins [16,17,18]—known as RNA interference [19,20,21]. miRNAs are involved in the regulation of 60–90% of protein-coding genes, affecting various biological processes, including development, differentiation, and metabolism, and have been implicated in the pathogenesis of various diseases, including ICH [21]. miRNAs are emerging as important regulators in the pathogenesis and treatment of various diseases, particularly malignant diseases such as cancer, in which miRNAs participate in initiation, progression, and metastasis. Abnormal miRNA expression has also been linked to the development and progression of cardiovascular and neurological disorders. Understanding the association between miRNAs and disease pathogenesis is crucial for improving diagnosis, treatment, and prognosis [22].

The association between miRNAs and ICH has been the subject of extensive recent research. miRNA dysregulation plays a crucial role in the pathogenesis and progression of ICH [23]. Through their regulation of mRNA translation [24], miRNAs can enhance the survival and neuroprotective capacity of mesenchymal stem cells in the treatment of ICH models [25]. miR-124, miR-340-5p, and miR-26a-5p play important roles in ICH [26,27,28]. These miRNAs have been associated with key biological processes, such as cell proliferation, differentiation, and metabolism, and their dysregulation can contribute to the development of ICH [29,30].

miR-195-5p participates in many diseases, including preeclampsia [31], inflammatory bowel disease [32], cerebral ischemia–reperfusion injury [33], colorectal cancer [34], and subarachnoid haemorrhage [35]. miR-195-5p is a critical regulator of many biological events, such as cell proliferation and invasion [36], oxidative stress [37] and tight junction redistribution [38], and it is a candidate diagnostic marker for various diseases [39,40]. Recent studies have shown that miR-195-5p has significant neuroprotective effects against ICH-induced brain damage, primarily through the inhibition of matrix metalloproteinases (MMP-9 and MMP-2), which are associated with secondary brain injury [41]. Treatment with miR-195-5p has been demonstrated to reduce brain edema, restore BBB integrity, and decrease neuronal apoptosis [8]. Accordingly, this study aimed to determine whether miR-195-5p exerts beneficial effects on brain tissue affected by ICH and elucidate the underlying mechanisms.

## 2. Results

### 2.1. miR-195-5p Prevents Neurological Dysfunction Following Intracerebral Hemorrhage in Rat Models

**Figure 1:**
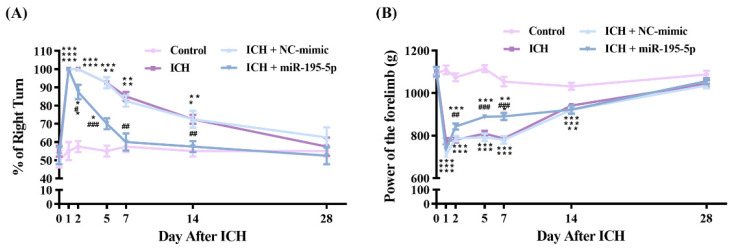
Neurobehavioral recovery in rats after ICH induction assessed by corner turn and grip strength tests. Over the 28-day period following ICH induction with collagenase, neurobehavior was compared between the control, ICH, ICH + NC-mimic, and ICH + miR-195-5p groups (*n* = 5). (**A**) Corner turn test showing the percentage of right turns made. Data were analyzed using one-way ANOVA followed by Tukey’s post hoc test to compare specific groups. (**B**) Grip test results showing grip strength as a percentage of the control group’s performance. Statistical analysis was performed using two-way ANOVA with Tukey’s post hoc test to assess differences between individual groups. * *p* < 0.05, ** *p* < 0.01, and *** *p* < 0.001 compared to control group. # *p* < 0.05, ## *p* < 0.01, and ### *p* < 0.001 compared between the ICH and miR-195-5p-treated groups.

We investigated the neuroprotective effects of miR-195-5p on brain damage following ICH using both the corner turn and forelimb grip strength tests as measures of neurological function. For the corner turn test, we performed a two-way ANOVA to evaluate the interaction between time and group, and both factors were found to be statistically significant (group: F = 22.770, *n* = 4, *p* < 0.01; time: F = 18.123, *n* = 7, *p* < 0.001). A significant interaction between time and group was also observed (F = 2.134, *p* < 0.01). The ICH group demonstrated a substantial increase in the percentage of right turns, in contrast with the performance of the control group on days 1, 2, 5, 7, and 14 (Figure 1A) (*n* = 5, *p* < 0.001 at day 1, 2, and 5; *p* < 0.01 at day 7 and 14). However, the miR-195-5p-treated rats showed significant neurobehavioral improvements, as evidenced by the increased percentage of right turns (Figure 1A) (*n* = 5, *p* < 0.05 at day 2; *p* < 0.01 at day 7 and 14; *p* < 0.001 at day 5), indicating a robust recovery in ICH-induced neurological deficits. Similarly, for the forelimb grip strength test, a two-way ANOVA also showed a significant effect for both time and group (Group: F= 176.451, *n* = 4, *p* < 0.01; Time: F = 119.517, *n* = 7, *p* < 0.001). Additionally, the interaction between group and time was significant (F = 15.683, *p* < 0.001). The forelimb grip strength test revealed that the ICH group experienced a notable reduction in gripping force compared to the controls at the same post-ICH intervals (Figure 1B) (*n* = 5, *p* < 0.001 at day 1, 2, 5, 7, and 14). Treatment with miR-195-5p was associated with a significant restoration of forelimb grip strength on days 2, 5, and 7 after ICH induction (Figure 1B) (*n* = 5, *p* < 0.01 at day 2; *p* < 0.001 at day 5 and 7). These findings underscore the therapeutic potential of miR-195-5p in ameliorating ICH-induced neural impairment.

### 2.2. Effect of miR-195-5p on Tissue Damage and Apoptosis Following Intracerebral Hemorrhage (ICH)

**Figure 2:**
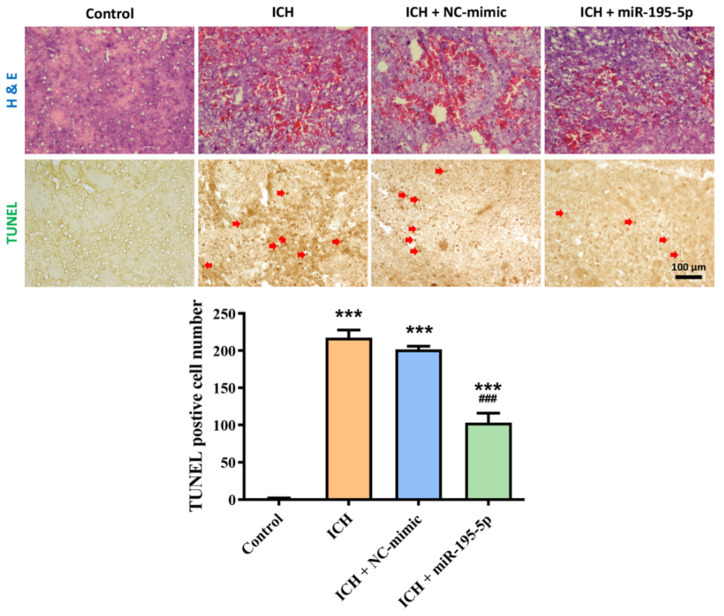
Effect of miR-195-5p on tissue damage and apoptosis following intracerebral hemorrhage (ICH) (*n* = 4 in each group). Representative images of brain sections stained with H&E (**top** row) and TUNEL (**bottom** row) from different experimental groups: Control, ICH, ICH + NC-mimic, and ICH + miR-195-5p. Red arrows in the TUNEL images indicate TUNEL-positive cells. The bar graph below shows the quantification of TUNEL-positive cells across groups, with data presented as mean ± standard deviation. *** *p* < 0.001 compared to the control group; ### *p* < 0.001 compared to the ICH group.

To evaluate the protective effects of miR-195-5p on brain tissue following intracerebral hemorrhage (ICH), we utilized H&E staining to assess tissue architecture and hemorrhage, and TUNEL staining to measure apoptosis (Figure 2). In the H&E-stained sections, the control group exhibited normal brain tissue with intact architecture, free of hemorrhage or damage. However, in the ICH group, there was a marked disruption of tissue structure accompanied by extensive hemorrhage, evidenced by the significant infiltration of red blood cells into the brain parenchyma. The ICH + NC-mimic group displayed a similar pattern of tissue damage and hemorrhage to the ICH group, suggesting that the NC-mimic treatment did not alleviate the effects of the hemorrhage. In contrast, the ICH + miR-195-5p group showed a noticeable reduction in tissue disruption and hemorrhage, indicating that miR-195-5p treatment may help to preserve tissue integrity following ICH. The TUNEL-stained images provide further insights into the effects of these treatments on apoptosis. The Kruskal–Wallis test results for the TUNEL staining showed significant differences in the number of TUNEL-positive cells between the experimental groups (H = 13.172, *n* = 4, *p* < 0.01). Specifically, the control group showed minimal TUNEL-positive cells (1.5 ± 1.29), reflecting a low level of apoptosis in healthy brain tissue. In the ICH group, there was a significant increase in TUNEL-positive staining (217.5 ± 20.49), indicating widespread apoptosis as a consequence of the hemorrhage (*n* = 4, *p* < 0.001). The ICH + NC-mimic group similarly showed high levels of apoptosis (201.5 ± 9.47), comparable to the untreated ICH group (*n* = 4, *p* < 0.001). However, the ICH + miR-195-5p group revealed a substantial reduction in TUNEL-positive cells (103.25 ± 25.38) (*n* = 4, *p* < 0.001), suggesting that miR-195-5p treatment effectively diminishes apoptosis in brain tissue following ICH. These results collectively suggest that miR-195-5p exerts protective effects by reducing both tissue damage and apoptosis in the brain after ICH, underscoring its potential therapeutic ability.

### 2.3. miR-195-5p Modulates Macrophage Polarization in Intracerebral Hemorrhage

**Figure 3:**
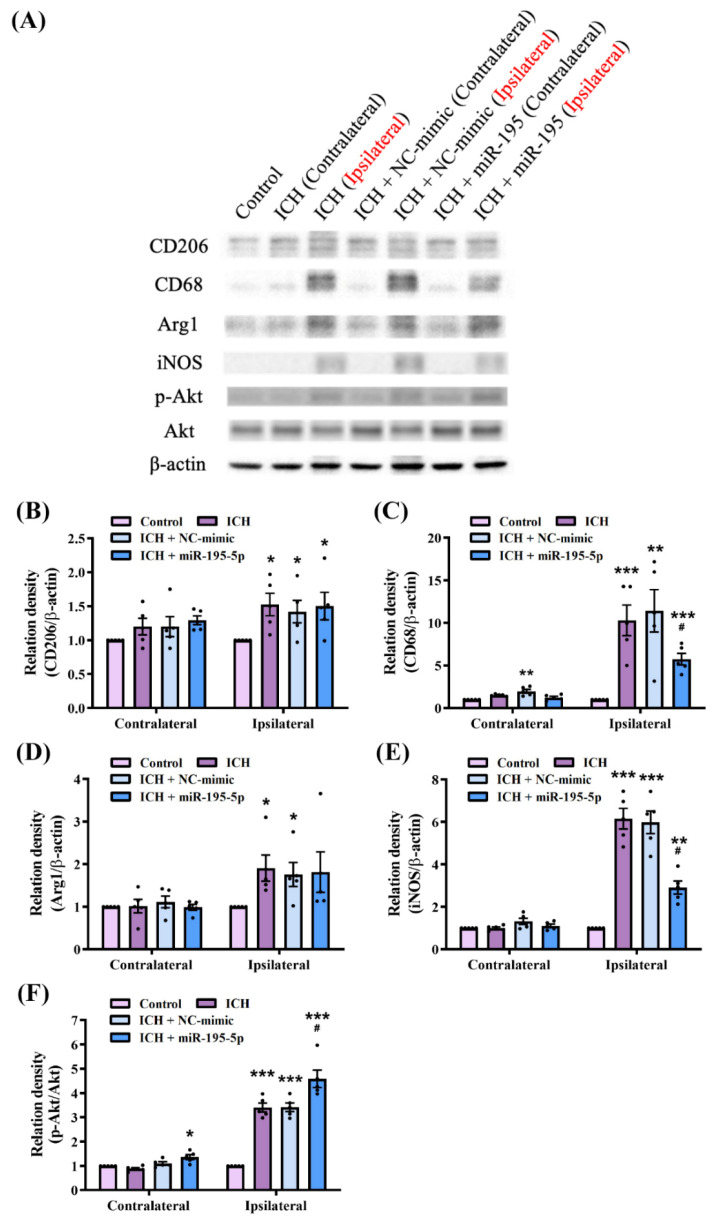
Effects of miR-195-5p on macrophage polarization and Akt signalling pathways following ICH (*n* = 5 in each group). (**A**) Western blot analysis of CD206, CD68, Arg1, iNOS, p-Akt, total Akt, and β-actin levels between experimental groups: control, ICH (contralateral and ipsilateral), ICH + NC-mimic (contralateral and ipsilateral), and ICH + miR-195-5p (contralateral and ipsilateral). β-actin was used as the loading control. Quantification of CD206 (**B**), CD68 (**C**), Arg1 (**D**), iNOS (**E**), and p-Akt/total Akt (**F**), protein level normalized to β-actin level. Data presented as mean ± standard deviation and were analyzed using Kruskal–Wallis one-way analysis of variance and Student *t* test for specific group comparisons. * *p* < 0.05, ** *p* < 0.01, and *** *p* < 0.001 compared to control group. # *p* < 0.05 compared between the ICH and miR-195-5p-treated groups.

We investigated the macrophage polarization effects of miR-195-5p following ICH based on the level of macrophage-polarization-related proteins detected by the western blotting (Figure 3A), including CD206, CD68, arginase 1 (Arg1), inducible nitric oxide synthase (iNOS), and Akt. CD68 is a marker of activated microglia and macrophages involved in the inflammatory response to brain injury [42]. iNOS produces nitric oxide, a reactive molecule that contributes to oxidative stress and inflammation [43]. CD206 and Arg1 are markers of alternatively activated (M2) macrophages that are associated with anti-inflammatory and tissue-repair processes [44]. Akt signalling, particularly the phosphorylation of Akt (p-Akt), is involved in macrophage polarization [45,46].

The results of the Kruskal–Wallis test quantifying the western blot data revealed significant differences in the expression levels of CD206 (H = 8.489, *n* = 5, *p* < 0.05), CD68 (H = 16.969, *n* = 5, *p* < 0.05), Arg1 (H = 9.772, *n* = 5, *p* < 0.05), iNOS (H = 13.152, *n* = 5, *p* < 0.05), and p-Akt/Akt (H = 14.660, *n* = 5, *p* < 0.01) between the experimental groups. The ICH group showed a significant increase in CD68 and iNOS expression in the ipsilateral hemisphere (CD68 and iNOS: *n* = 5, *p* < 0.001), indicating a heightened inflammatory response. This increase was notably mitigated in the miR-195-5p-treated group (CD68 and iNOS: *n* = 5, *p* < 0.05) (Figure 3A,C,E), suggesting that miR-195-5p exerts anti-inflammatory effects. Interestingly, the levels of CD206 and Arg1 did not decrease following the miR-195-5p treatment (CD206 and Arg1: *n* = 5, *p* < 0.001) (Figure 3A,B,D). These results indicate that miR-195-5p does not influence the increase in the M2 phenotype. However, the significant increase in the ratio of p-Akt to total Akt observed in the miR-195-5p-treated group suggests that macrophages were activated (p-Akt/Akt: *n* = 5, *p* < 0.05) (Figure 3A,F). Upon further examination of iNOS and other inflammation-related proteins, it was found that their levels were reduced after the miR-195-5p treatment, indicating that miR-195-5p may inhibit the activation of the M1 phenotype rather than directly promoting M2 polarization. These molecular changes were more pronounced in the ipsilateral than in the contralateral hemisphere, emphasizing the localized response to injury and treatment. These findings suggest that miR-195-5p modulates macrophage polarization by inhibiting M1 activation in response to ICH, potentially contributing to neuroprotection and neural function recovery.

### 2.4. miR-195-5p Modulates Oxidative Stress and Inflammatory Pathways Following Intracerebral Hemorrhage

**Figure 4:**
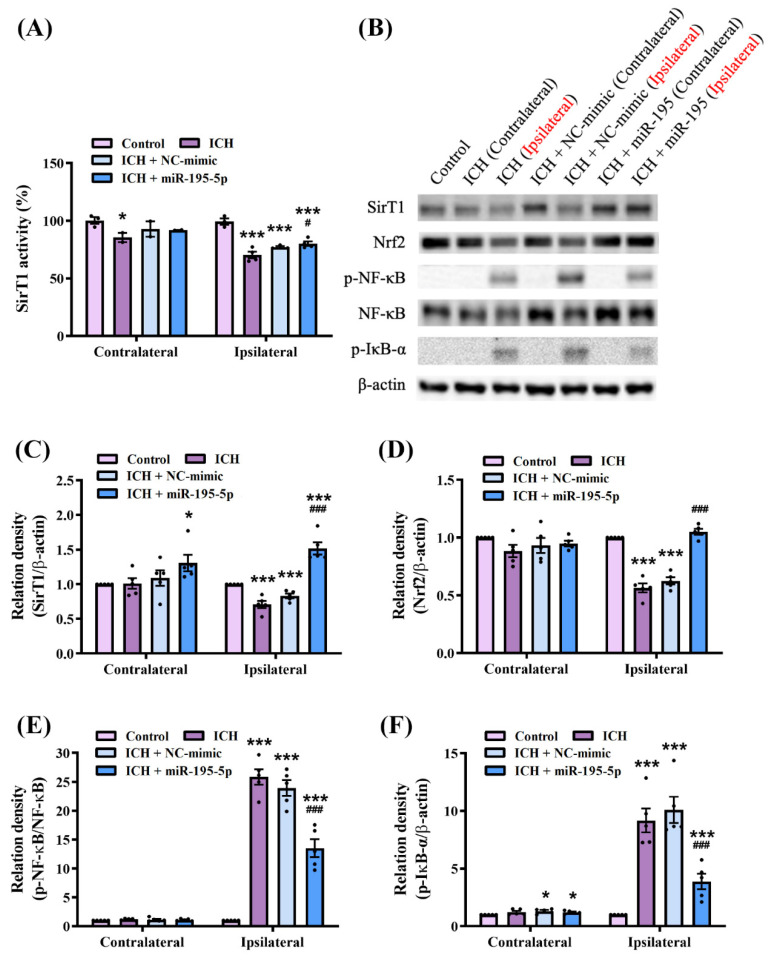
miR-195-5p modulates SirT1 activity, oxidative stress, and inflammatory pathways in response to intracerebral hemorrhage (*n* = 5 in each group). (**A**) SirT1 activation assay. (**B**) Western blot analysis showing the levels of Sirt1, Nrf2, p-NF-κB, total NF-κB, p-IκB-α, and β-actin between experimental groups: control, ICH (contralateral and ipsilateral), ICH + NC-mimic (contralateral and ipsilateral), and ICH + miR-195-5p (contralateral and ipsilateral). β-actin was used as the loading control. Quantification of Sirt1 (**C**), Nrf2 (**D**), p-NF-κB/total NF-κB (**E**), and p-IκB-α (**F**), protein level normalized to β-actin level. Data presented as mean ± standard deviation and were analyzed using the Kruskal–Wallis one-way analysis of variance and Student t test for specific group comparisons. * *p* < 0.05 and *** *p* < 0.001 compared to control group. # *p* < 0.05 and ### *p* < 0.001 compared between the ICH and miR-195-5p-treated groups.

To assess the deacetylation activity of SirT1, we employed a SirT1 activity assay (Figure 4A). The results of the Kruskal–Wallis test for SirT1 activity revealed significant differences in the different groups (H = 17.721, *n* = 5, *p* < 0.01). The results revealed a significant reduction in SIRT1 activity in the ipsilateral hemisphere of the ICH group compared to the control group (*n* = 5, *p* < 0.001). This decrease in activity was similarly observed in the ICH + NC-mimic group. However, in the ICH + miR-195-5p-treated group, SirT1 activity was significantly restored in the ipsilateral hemisphere compared to the ICH group (*n* = 5, *p* < 0.05) (Figure 4B). In addition, we investigated the molecular mechanisms underlying the neuroprotective effects of miR-195-5p following ICH based on the expression of key proteins involved in oxidative stress and inflammatory pathways (Figure 4B), including SirT1, Nrf2, phosphorylated NF-κB (p-NF-κB), and NF-κB. The results of the Kruskal–Wallis test quantifying the western blot data revealed significant differences in the expression levels of SirT1 (H = 9.956, *n* = 5, *p* < 0.05), Nrf2 (H = 12.0.38, *n* = 5, *p* < 0.01), p-NF-κB/NF-κB (H = 13.166, *n* = 5, *p* < 0.01), and p-IκB-α (H = 13.701, *n* = 5, *p* < 0.01) between the experimental groups. Western blotting showed that the level of Sirt1, a biomarker of cellular stress resistance and longevity, increased significantly in the ipsilateral hemisphere following the miR-195-5p treatment compared to those in both the control and the ICH group (control and IHC: *n* = 5, *p* < 0.001), suggesting that miR-195-5p enhances cellular resilience to ICH-induced stress (*n* = 5, *p* < 0.001) (Figure 4C). The level of Nrf2, a biomarker of antioxidant responses, was significantly downregulated following ICH, indicating compromised antioxidant defence (*n* = 5, *p* < 0.001). However, the miR-195-5p treatment restored Nrf2 levels, highlighting its potential for bolstering the antioxidant capacity of brain tissue post-ICH (*n* = 5, *p* < 0.001) (Figure 4D). NF-κB, a transcription factor that plays a pivotal role in inflammation, was activated in the ICH group, as evidenced by increased levels of p-NF-κB (*n* = 5, *p* < 0.001). This activation was significantly attenuated by the miR-195-5p treatment (*n* = 5, *p* < 0.001), demonstrating its anti-inflammatory properties (Figure 4E). Additionally, the phosphorylation of IκB-α, which regulates NF-κB activity, was elevated in the ICH group (*n* = 5, *p* < 0.001) but reduced following miR-195-5p treatment, further supporting the anti-inflammatory effect of miR-195-5p (*n* = 5, *p* < 0.001) (Figure 4F). These findings suggest that miR-195-5p exerts its neuroprotective and neuroregenerative effects by modulating critical oxidative stress and inflammatory pathways.

### 2.5. miR-195-5p Attenuated Intracerebral Hemorrhage-Induced Inflammation Following Intracerebral Hemorrhage Induction

**Figure 5:**
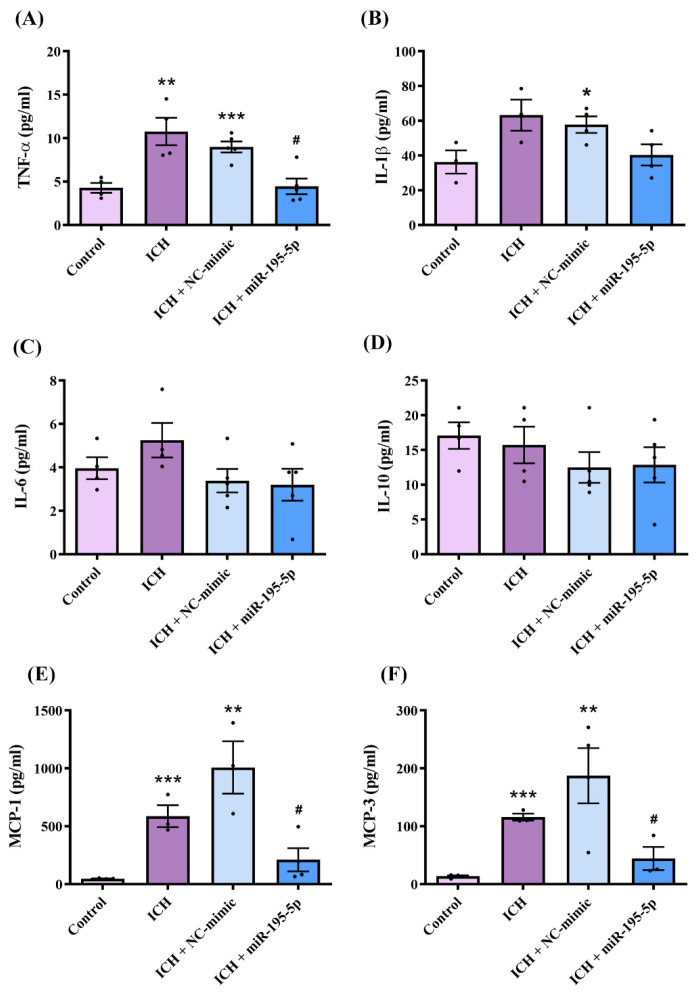
Effects of miR-195-5p on inflammatory cytokine levels following intracerebral hemorrhage (*n* = 4 in each group). The levels of TNF-α (**A**), IL-1β (**B**), IL-6 (**C**), IL-10 (**D**), MCP-1 (**E**), and MCP-3 (**F**) were measured using ELISA in the control, ICH, ICH + NC-mimic, and ICH + miR-195-5p groups. Data presented as mean ± standard deviation and were analyzed using the Kruskal–Wallis one-way analysis of variance and Student *t* test for specific group comparisons. * *p* < 0.05, ** *p* < 0.01, and *** *p* < 0.001 compared to control group. # *p* < 0.05 compared between the ICH and miR-195-5p-treated groups.

We elucidated the anti-inflammatory potential of miR-195-5p following ICH induction based on the quantitative expression of key pro-inflammatory and anti-inflammatory markers at the lesion site detected by ELISA, including TNF-α, IL-1β, IL-6, IL-10, MCP-1, and MCP-3 (Figure 5A–F). Based on the Kruskal–Wallis test for the ELISA results, the levels of MCP-1 (H = 11.490, *n* = 4, *p* < 0.01) and MCP-3 (H = 10.676, *n* = 4, *p* < 0.01) showed significant differences between the experimental groups. However, there were no statistically significant differences in the levels of IL-1β (H = 6.782, *n* = 4, *p* = 0.079), IL-10 (H = 2.971, *n* = 4, *p* = 0.396), IL-6 (H = 4.660, *n* = 4, *p* = 0.198), and TNF-α (H = 1.292, *n* = 4, *p* = 0.731) across the groups. There was a marked upregulation in TNF-α, IL-1β, MCP-1, and MCP-3 levels in the ICH and ICH + NC-mimic groups compared to those in the control group, reflecting the robust inflammatory response elicited by ICH (with the exception of IL-1β, which did not show a significant difference in the ICH group). Crucially, miR-195-5p administration significantly downregulated TNF-α (*n* = 4, *p* < 0.05), MCP-1 (*n* = 4, *p* < 0.05), and MCP-3 (*n* = 4, *p* < 0.05) levels to below those observed in the ICH group. These results indicate the potent anti-inflammatory effects of miR-195-5p and support its potential as a therapeutic agent for attenuating the inflammatory aftermath of ICH.

### 2.6. miR-195-5p Modulates the Apoptosis Mechanism in Intracerebral Hemorrhage

**Figure 6:**
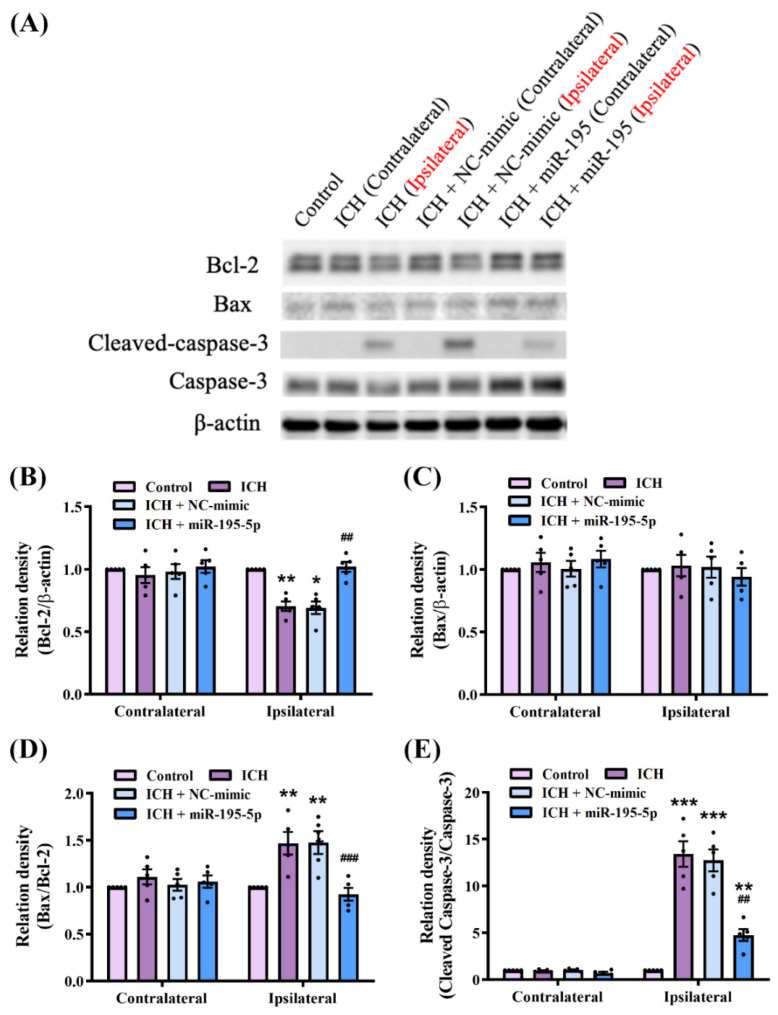
Impact of miR-195-5p on apoptosis-related protein level in the brain following intracerebral hemorrhage (*n* = 5 in each group). (**A**) Western blot analysis showing Bcl-2, Bax, cleaved caspase-3, caspase-3, and β-actin levels between groups: control, ICH (contralateral and ipsilateral), ICH + NC-mimic (contralateral and ipsilateral), and ICH + miR-195-5p (contralateral and ipsilateral). Bcl-2 (**B**), Bax (**C**), Bax/Bcl2 (**D**), and cleaved caspase-3/caspase-3 (**E**), level of proteins normalized to β-actin level. Data presented as mean ± standard deviation and were analyzed using the Kruskal–Wallis one-way analysis of variance and Student *t* test for specific group comparisons. * *p* < 0.05, ** *p* < 0.01 and *** *p* < 0.001 compared to control group. ## *p* < 0.01 and ### *p* < 0.001 compared between the ICH and miR-195-5p-treated groups.

To confirm the apoptotic mechanism associated with miR-195-5p treatment in ICH, we compared the protein levels of Bcl-2, Bax, and caspase-3 between the control, ICH, ICH + NC-mimic, and ICH + miR-195-5p groups by western blotting (Figure 6A). The results of the Kruskal–Wallis test quantifying the western blot data revealed significant differences in the expression levels of Bcl2 (H = 10.110, *n* = 5, *p* < 0.01), cleaved caspase-3/caspase-3 (H = 11.377, *n* = 5, *p* < 0.05), and Bax/Bcl2 (H = 9.706, *n* = 5, *p* < 0.05) between the experimental groups. In the contralateral hemisphere, there were no significant differences in protein level among the groups, indicating the localized effect of ICH on the ipsilateral or injured hemisphere. However, on the ipsilateral side, ICH reduced the level of Bcl-2, the anti-apoptotic protein (*n* = 5, *p* < 0.01), and increased the ratio of cleaved caspase-3 to caspase-3 (*n* = 5, *p* < 0.001), suggesting enhanced apoptotic activity. There was no difference in Bax level between the groups in either hemisphere (Figure 6B–D). Remarkably, the administration of miR-195-5p reversed ICH-induced changes on the ipsilateral side. The miR-195-5p treatment significantly increased Bcl-2 levels (*n* = 5, *p* < 0.01), suggesting a reduction in apoptosis. Additionally, the miR-195-5p treatment decreased the ratio of cleaved caspase-3 to caspase-3 (*n* = 5, *p* < 0.01), further indicating the suppression of apoptosis (Figure 6B–D). These findings support the role of miR-195-5p in modulating apoptosis-related proteins and potentially reducing apoptosis in brain tissue affected by ICH.

### 2.7. miR-195-5p Modulates Synaptic Plasticity Pathways in Response to Intracerebral Hemorrhage

**Figure 7:**
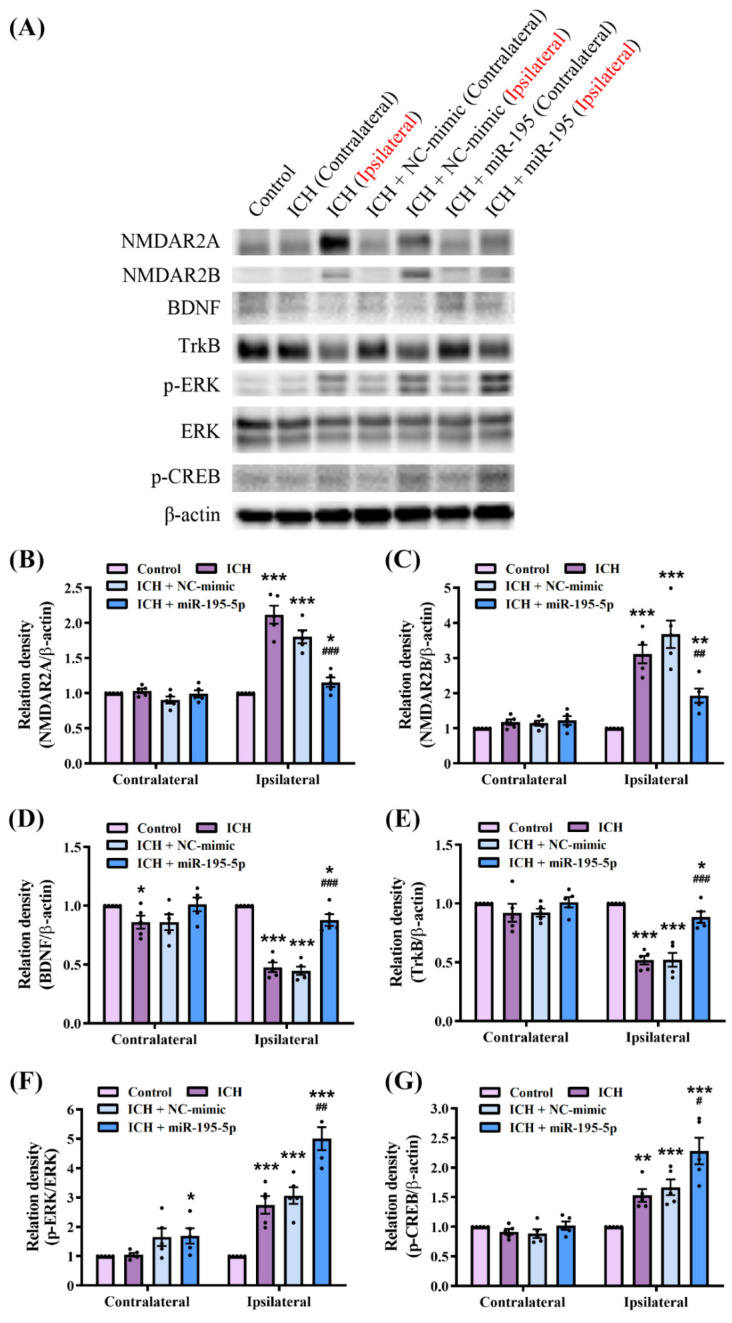
Impact of miR-195-5p on synaptic-plasticity-related protein level in the brain following intracerebral hemorrhage (*n* = 5 in each group). (**A**) Western blot analysis of NMDAR2A, NMDAR2B, BDNF, TrkB, p-ERK, ERK, p-CREB, and β-actin levels between groups: control, ICH (contralateral and ipsilateral), ICH + NC-mimic (contralateral and ipsilateral), and ICH + miR-195-5p (contralateral and ipsilateral). Quantification of NMDAR2A (**B**), NMDAR2B (**C**), BDNF (**D**), TrkB (**E**), p-ERK/ERK (**F**), and p-CREB (**G**), protein levels normalized to β-actin levels. Data presented as mean ± standard deviation and were analyzed using the Kruskal–Wallis one-way analysis of variance and Student *t* test for specific group comparisons. * *p* < 0.05, ** *p* < 0.01 and *** *p* < 0.001 compared to control group. # *p* < 0.05, ## *p* < 0.01 and ### *p* < 0.001 compared between the ICH and miR-195-5p-treated groups.

We investigated the mechanisms underlying the neuroprotective effects of miR-195-5p in ICH by comparing the NMDAR2A, NMDAR2B, BDNF, TrkB, ERK, and phosphorylated CREB (p-CREB) levels between the control, ICH, ICH + NC-mimic, and ICH + miR-195-5p groups by western blotting (Figure 7A). The results of the Kruskal–Wallis test quantifying the western blot data revealed significant differences in the expression levels of NMDAR2A (H = 13.535, *n* = 5, *p* < 0.01), NMDAR2B (H = 12.862, *n* = 5, *p* < 0.01), BDNF (H = 12.839, *n* = 5, *p* < 0.01), TrkB (H = 12.908, *n* = 5, *p* < 0.01), p-ERK/ERK (H = 12.248, *n* = 5, *p* < 0.01), and p-CREB (H = 13.008, *n* = 5, *p* < 0.05) between the experimental groups. In the ipsilateral hemisphere, ICH induced a significant increase in the levels of NMDAR2A and NMDAR2B compared to those in the control group. These proteins are subunits of NMDA receptors involved in synaptic transmission and plasticity (NMDAR2A: *n* = 5, *p* < 0.001; NMDAR2B: *n* = 5, *p* < 0.001). Treatment with miR-195-5p significantly reduced the levels of these proteins compared to those in the ICH group (NMDAR2A: *n* = 5, *p* < 0.001; NMDAR2B: *n* = 5, *p* < 0.01) (Figure 7B,C). Additionally, the BDNF level was significantly lower in the ipsilateral hemisphere of the ICH group than in the control group (*n* = 5, *p* < 0.001). However, the treatment with miR-195-5p significantly increased BDNF levels compared to those in the ICH group (*n* = 5, *p* < 0.001) (Figure 7D). The TrkB levels showed the same pattern (*n* = 5, *p* < 0.001) (Figure 7E). The levels of p-ERK/ERK (*n* = 5, *p* < 0.001) and p-CREB (*n* = 5, *p* < 0.01) were significantly higher in the ipsilateral hemisphere in the ICH group than those in the control group, which were significantly reduced by the miR-195-5p treatment (p-ERK/ERK: *n* = 5, *p* < 0.01; p-CREB: *n* = 5, *p* < 0.05) (Figure 7F,G). These molecular changes were more notable in the ipsilateral than in the contralateral hemisphere, highlighting the localized response to injury and treatment. These findings suggest that miR-195-5p modulates the level of synaptic-plasticity-related proteins, potentially mitigating ICH-induced synaptic dysfunction. Collectively, these findings suggest that miR-195-5p may have a beneficial effect on neuronal signalling pathways under ICH, potentially aiding in the recovery of neural function.

## 3. Discussion

In our study, the ICH rats exhibited significant neurological deficits due to increased apoptosis, as evidenced by changes in the activity of apoptotic markers, such as the elevated cleaved caspase-3–caspase-3 ratio and reduced Bcl-2 level. Nevertheless, while the Bcl-2 levels decreased following ICH, the Bax levels remained unchanged, suggesting that the apoptotic response in this model is primarily driven by the modulation of anti-apoptotic factors like Bcl-2 rather than changes in Bax. These molecular changes led to substantial impairments in neurological function, as demonstrated by the corner turn and forelimb grip strength tests, in which the ICH rats showed increased right turns and decreased forelimb strength. However, the miR-195-5p treatment effectively rescued neurological function by reducing apoptosis, as indicated by the increased Bcl-2 level and decreased cleaved caspase-3–caspase-3 ratio, leading to a marked recovery in both directional turning and forelimb strength. Collectively, these findings suggested that miR-195-5p promotes robust recovery from ICH-induced deficits.

The inflammatory aftermath of ICH involves the upregulation of cytokines and chemokines that mobilize immune cells to the central nervous system (CNS) parenchyma [47]. A key component of this process is the recruitment of neutrophils and monocyte-derived cells, including microglia, facilitated by CXC and CC chemokines [48]. Among these, monocyte chemotactic proteins (MCPs), particularly MCP-1 (CCL2) and MCP-3 (CCL7), play important roles in regulating immune cell trafficking via the CCR2 receptor [49]. The levels of MCP-1, which are low under normal conditions, dramatically increase following CNS injury, creating a level gradient that attracts CCR2-expressing monocytes and microglia to the injury site [50]. Recruited immune cells contribute to secondary injury by releasing cytokines, chemokines, and other reactive species [51]. Microglia, the resident immune cells of the CNS, undergo phenotypic changes after injury: they shift from a surveillance state to an activated state, characterized by changes in morphology, alterations in gene expression, and migration to the injury site [52]. Activated microglia can adopt either a pro-inflammatory (M1) or an anti-inflammatory (M2) phenotype. The M1 phenotype is associated with the production of proinflammatory cytokines and reactive oxygen species, exacerbating tissue damage, whereas the anti-inflammatory M2 phenotype is involved in tissue repair [53,54]. In our study, the ICH rats showed increased pro-inflammatory marker (CD68 and iNOS) levels and decreased anti-inflammatory marker (CD206 and Arg1) levels. The miR-195-5p treatment inhibited M1-type macrophage polarization, reducing CD68 and iNOS levels. Additionally, Akt signalling, which is crucial for macrophage polarization and tissue repair, was amplified in the miR-195-5p-treated group. The ELISA assays showed that ICH significantly upregulated pro-inflammatory cytokine (TNF-α, MCP-1, and MCP-3) expression. The miR-195-5p treatment significantly reduced the levels of these cytokines, indicating the potent attenuation of the ICH-induced inflammatory response. These results indicate the potent anti-inflammatory effects of miR-195-5p and support its potential as a therapeutic agent for attenuating the inflammatory aftermath of ICH. However, the expression of IL-10, an anti-inflammatory cytokine [55], did not show any significant differences. This suggests that miR-195-5p primarily inhibits the differentiation of macrophages into the M1 phenotype, rather than promoting M2 differentiation, which is more closely associated with anti-inflammatory effects. Consequently, miR-195-5p’s actions are more related to suppressing inflammation than to enhancing anti-inflammatory responses. Oxidative stress and inflammatory pathways are also crucial in ICH [56]. Sirt1 is a NAD+-dependent deacetylase that plays a pivotal role in cellular stress resistance and longevity [57]. It exerts neuroprotective effects by deacetylating and thereby inhibiting the activity of NF-κB, a key transcription factor that promotes the expression of pro-inflammatory genes [58]. When NF-κB is activated, it translocates to the nucleus and induces the expression of various pro-inflammatory cytokines, such as TNF-α, IL-1β, and IL-6, which can exacerbate neuronal damage [58]. The Sirt1-mediated deacetylation of the p65 subunit of NF-κB reduces its transcriptional activity, leading to decreased inflammation and reduced neuronal apoptosis [58]. Nrf2, another crucial regulator, is a transcription factor that controls the expression of antioxidant proteins that protect against oxidative damage triggered by injury and inflammation [59]. Under normal conditions, Nrf2 is bound to Keap1 in the cytoplasm and is rapidly degraded. However, under oxidative stress, Nrf2 is released from Keap1, translocates to the nucleus, and binds to the antioxidant response element (ARE) to initiate the transcription of antioxidant genes like HO-1 and NQO1 [60,61,62]. In the context of ICH, oxidative stress activates NF-κB, which in turn increases the production of inflammatory mediators and exacerbates tissue damage [63]. Sirt1 counters this by deacetylating NF-κB, thereby reducing its pro-inflammatory effects. Simultaneously, Sirt1 also enhances the activity of Nrf2, promoting the expression of antioxidant enzymes that mitigate oxidative stress [59]. The phosphorylation of IκB-α (p-IκB-α) plays a role in NF-κB activation, as p-IκB-α leads to the degradation of IκB-α, freeing NF-κB to enter the nucleus and activate inflammatory genes [64]. Thus, Sirt1’s inhibition of NF-κB through deacetylation can also be understood as indirectly reducing IκB-α phosphorylation, further dampening the inflammatory response. We found that ICH downregulated Sirt1 and Nrf2 levels, compromising cellular resilience and antioxidant defences, and activated NF-κB, promoting inflammation. The miR-195-5p treatment enhanced Sirt1 and Nrf2 levels, bolstering cellular resilience and antioxidant capacity, while concurrently attenuating NF-κB activation, thereby reducing inflammation. The transcription factor NF-κB, which is sensitive to oxidative stress, particularly in perihematomal regions under ICH, mediates the induction of key genes in the CNS inflammatory response [13,65]. NF-κB activation promotes the expression of enzymes and cytokines such as IL-1β, which modulate immune responses, and promotes BBB permeability and edema. This cascade not only drives inflammation, but also promotes apoptosis and amplifies cellular damage [66]. The inflammatory response, driven by cytokines and chemokines such as MCP-1, exacerbates neuronal injury and cell death through oxidative stress and exacerbated cytokine release. Apoptosis is further induced by the proinflammatory milieu, with NF-κB playing a central role [67]. Therefore, targeting the MCP-1–CCR2 axis and modulating microglial activation states (M1/M2) are promising strategies for mitigating inflammation and apoptosis and improving treatment outcomes in ICH [68].

ICH disrupts synaptic plasticity and function, leading to cognitive and motor deficits. NMDAR2A, NMDAR2B, BDNF, TrkB, ERK, and CREB are key proteins in the maintenance of neuronal communication and plasticity. Alterations in the expression and activity of these proteins exacerbate neuronal damage and hinder recovery [69]. NMDAR2A and NMDAR2B are subunits of the NMDA receptor, which plays a crucial role in synaptic plasticity and neuroprotection [70]. The activation of NMDA receptors leads to the influx of calcium ions, which can then trigger a series of intracellular signaling cascades. One significant pathway involves the activation of brain-derived neurotrophic factor (BDNF) and its receptor, TrkB. BDNF binding to TrkB activates downstream signaling molecules, including ERK (extracellular signal-regulated kinase) and CREB (cAMP response element-binding protein), both of which are crucial for synaptic plasticity and neuronal survival. The ERK pathway, once activated, can phosphorylate CREB, a transcription factor that promotes the expression of genes involved in neuroprotection and synaptic plasticity, such as BDNF itself [71,72]. This creates a positive feedback loop in which BDNF enhances its own expression via TrkB, ERK, and CREB. Our results demonstrated that ICH significantly upregulated the expression of the NMDAR2A and NMDAR2B subunits of NMDA receptors, which are critical for synaptic transmission and plasticity. The overexpression of these subunits following ICH probably contributes to excitotoxicity, which reduces neuronal survival. The miR-195-5p treatment effectively reduced the levels of NMDAR2A and NMDAR2B, suggesting a protective role against excitotoxic damage. BDNF and its receptor, TrkB, are crucial for neuronal survival, differentiation, and synaptic plasticity. Our findings revealed that ICH significantly decreased BDNF and TrkB levels, potentially impairing neuroplasticity and recovery, whereas the miR-195-5p treatment reversed these changes in protein level. The upregulation of BDNF and TrkB facilitates neuroprotection and synaptic repair, thereby promoting functional recovery under ICH. Additionally, the levels of p-ERK/ERK and p-CREB, which are involved in cellular signalling and synaptic plasticity, were significantly elevated following ICH, which may reflect an initial neuronal repair or maladaptive response that contributes to the pathology. The miR-195-5p treatment normalized these phosphorylation levels, effectively restoring neuronal function. The localized response to the miR-195-5p treatment in the ipsilateral hemisphere underscores the specificity and potential safety of miR-195-5p in targeting the injury-induced dysregulation of synaptic-plasticity-related proteins. By modulating these proteins, miR-195-5p appears to mitigate synaptic dysfunction, reduce excitotoxicity, and enhance neurotrophic support, all of which are crucial for the recovery of neural function following ICH. The limitation of our study is the use of relatively simple behavioral tests, such as the corner turn test and forelimb grip strength test, to assess motor function recovery following ICH. While these tests provide valuable insights into gross motor impairments, they may not fully capture the complexity of motor deficits or the subtleties of functional recovery in ICH models. More comprehensive motor function assessments, such as the cylinder test, beam walking test, or ladder rung walking test, could offer a more nuanced understanding of motor coordination, balance, and fine motor skills. Future studies should incorporate these more appropriate and sensitive motor function tests to better evaluate the therapeutic potential of miR-195-5p in promoting functional recovery after ICH. Additionally, another limitation of the study is that we did not directly assess whether specific proteins are deacetylated by SIRT1 after miR-195-5p administration, although we observed elevated SIRT1 protein levels. Future research should employ SIRT1 inhibitors or deacetylation-specific assays to confirm the deacetylation of target proteins by SIRT1, providing further clarity on the molecular mechanisms underlying the neuroprotective effects of miR-195-5p.

## 4. Materials and Methods

### 4.1. Design, Model, and Surgical Preparations: Induction of Intracerebral Hemorrhage

**Figure 8:**
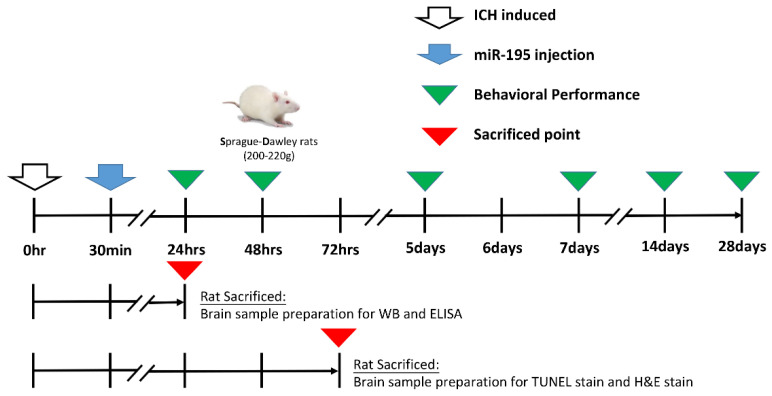
Experimental timeline and procedures for evaluating the effects of miR-195-5p on Intracerebral Hemorrhage (ICH) in Rats. ICH was induced at the 0 h mark, followed by the injection of miR-195-5p 30 min post-ICH. Behavioral performance assessments were conducted at multiple time points: 24 h, 48 h, 72 h, 5 days, 7 days, 14 days, and 28 days post-ICH. Rats were sacrificed at two different time points for tissue analysis. At 24 h post-ICH, brain samples were collected for western Blot (WB) and ELISA analyses. At 7 days post-ICH, rats were sacrificed for brain sample preparation for TUNEL staining and H&E staining to assess tissue damage and apoptosis.

We used male Sprague–Dawley rats (*N* = 60, 7 weeks old, 200–250 g). The animals had ad libitum access to food and water. All experimental procedures were approved by the Center for Laboratory Animals and Use Committee of Kaohsiung Medical University (approval no. 104236). Anaesthesia was achieved via an intraperitoneal injection of pentobarbital (50 mg/kg), and a heating pad (Harvard Apparatus) was used to maintain the rectal temperature at 36 ± 1 °C. We divided the rats into four groups: (1) control group with sham surgery, (2) ICH only, (3) ICH with NC-mimic treatment, and (4) ICH with miR-195-5p treatment. ICH was experimentally induced using a stereotaxic intrastriatal injection of type IV bacterial collagenase (Sigma-Aldrich; C5138; St. Louis; State of Missouri; USA) as previously described [73,74]. Briefly, after anesthetizing the rats with pentobarbital, they were placed in a stereotaxic apparatus (David Kopf Instruments, Tujunga, CA, USA). Burr holes were drilled into the skull and a 30-gauge Hamilton syringe needle was inserted into the striatum (located 3.0 mm lateral to the midline on the right, 0.2 mm posterior to the bregma, and 6 mm deep below the skull). ICH was induced by injecting 1 μL of a solution containing 0.23 μL type IV collagenase over 5 min. After infusion, the burr holes were sealed with bone wax, and the rats were housed individually for recovery. Following ICH induction, a dose of 5 nmol/mL/kg miR-195-5p (5′-UAGCAGCACAGAAAUAUUGGC-3′) was administered intravenously via the tail vein using in vivo jetPEI (Polyplus transfection; Illkirch; France). The experiment design is shown Figure 8.

### 4.2. Neurological Tests

We assessed the changes in neurological performance using corner turn and forelimb grip strength tests. 

#### 4.2.1. Corner Turn Test

The corner turn test was used to evaluate motor asymmetry and functional deficits following intracerebral hemorrhage (ICH). In this test, the rats were gently directed to a corner set at an angle of 30°. To exit the corner, the rats had the option of turning either left or right. The direction of the turn reflects the degree of asymmetry in motor function, with a preference for turning towards the injured side indicating greater neurological impairment. We recorded the percentage of rightward turns, focusing on asymmetry by noting the rat’s tendency to turn away from its injured side. For consistency, only turns made when the rat was fully reared against a wall at the start of the trial were considered. This test was conducted 10 times for each rat, with a minimum interval of 30 s between trials. The data are expressed as the percentage of rightward turns out of the total number of trials, providing a quantitative measure of motor asymmetry and functional outcome.

#### 4.2.2. Forelimb Grip Strength Test

The forelimb grip strength test was conducted at 1, 2, 5, 7, 14, and 28 d after ICH. Gripping force (g) was measured using a grip strength meter (BIO-GS3, BioSeb; Canada; USA). Briefly, the forelimb of each rat was gently placed on a grip bar and the rat was pulled horizontally until the bar was released. Measurements were made by an observer blinded to the treatment groups. Grip strength was normalized to rat body weight and expressed as a percentage of the control group values.

### 4.3. TUNEL and H&E Stain

Three days after the operation, rats from each group were re-anesthetized, and cardiac perfusion was performed using 250 mL of cold saline, followed by 250 mL of 4% paraformaldehyde in 0.1 mol/L phosphate-buffered saline (PBS). The brains were then fixed in 4% paraformaldehyde for 24 h and subsequently immersed in 30% sucrose for cryoprotection for another 24 h. After cryoprotection, the brains were sectioned into 12 μm slices using a cryostat, following the protocol described in previous studies. For in situ detection of DNA fragmentation, the brain sections were subjected to TUNEL staining using the in situ cell death detection kit (Roche, Basel, Switzerland). Additionally, hematoxylin and eosin (H&E) staining was performed to assess tissue morphology. Quantitative analysis of the stained cells was conducted using ImageJ software (version 1.51e, National Institutes of Health).

### 4.4. Western Blot Analysis

To investigate the role of miR-195-5p in ICH-induced injury, we assessed the protein levels by western blotting. At 24 h post-operation, rats were euthanized by decapitation and their brains were promptly removed (*n* = 6/group). Homogenates of the ipsilateral (ICH-affected tissue) and contralateral (unaffected tissue) hemispheres were separately centrifuged at 13,000 rpm for 20 min. Subsequently, 30 μg protein extract was electrophoresed on either 8% or 10% SDS-PAGE gels and then transferred onto PVDF membranes that were blocked with 5% skimmed milk in TBST (containing 50 mM Tris pH 7.5, 0.15 mM NaCl, and 0.05% Tween 20) and subsequently incubated with antibodies (Table S1) in TBST at 4 °C overnight. The membranes were incubated with horseradish-peroxidase-conjugated secondary antibodies in TBST for 1 h at room temperature. An anti-β-actin antibody served as the internal control. The bands were visualized using enhanced chemiluminescence (PerkinElmer; Waltham; MA; USA). The resulting blots were digitally captured (MiniChemi 500, Sagecreation; Beijing; China) and quantified using ImageJ software (National Institutes of Health, Bethesda, MD, USA).

### 4.5. ELISA

At 24 h after ICH induction, brain tissue samples were collected for qualitative and quantitative analyses of neuroinflammatory cytokines, including TNF-α, IL-1β, IL-6, IL-10, MCP-1, and MCP-3, using ProcartaPlex™ Immunoassay kits (ThermoFisher Scientific, ProcatrPlex Mix&Match Rat 6 plex, PPX-06-MX2W7TZ, Waltham, MA, USA) according to the manufacturer’s instructions.

### 4.6. SirT1 Enzymatic Activity Assay

The in vitro SirT1 enzymatic activity was assessed using a fluorometric assay kit from BPS Bioscience (Catalog #50081, Fountain Plaza, NY, USA). The assay setup involved pre-incubating the test compound with the SIRT enzyme, 5 µg of BSA, and the appropriate SirT assay buffer at room temperature for 30 min. To initiate the enzymatic reaction, the SIRT substrate was added to achieve a final concentration of 10 µM. The reaction continued for 30 min at 37 °C, followed by the addition of 2x SirT Developer to stop the reaction, and the mixture was incubated for an additional 15 min at room temperature. Fluorescence was then recorded using a Multimode Plate Reader (PerkinElmer VICTOR™ X2; Waltham; MA; USA) with excitation/emission wavelengths of 355/460 nm.

### 4.7. Statistical Analysis

Sample size was estimated based on previous protocols and data available in the literature [41]. Continuous values are presented as the mean ± standard deviation. For the neurological tests, a two-way ANOVA by Tukey’s post hoc test was employed to analyze group differences, while the results from western blot, TUNEL, and ELISA were analyzed using the Kruskal–Wallis one-way analysis of variance and Student *t* test (SPSS version 20.0, IBM SPSS, Armonk, NY, USA). Statistical significance was set at *p* < 0.05.

## 5. Conclusions

miR-195-5p exhibited comprehensive neuroprotective properties in our rat model of ICH and facilitated behavioral recovery by modulating the inflammatory response, oxidative stress, apoptosis, and synaptic plasticity. miR-195-5p is a promising therapeutic agent for improving the outcomes of patients with ICH by targeting multiple pathways involved in brain injury and recovery. Further research is warranted to explore its clinical applicability.

## Data Availability

Data are contained within the article.

## Supplementary Materials

The following supporting information can be downloaded at: https://www.mdpi.com/article/10.3390/ijms251910321/s1.
