# Supplementary material for: MicroRNA-195-5p Inhibits Intracerebral Hemorrhage-Induced Inflammatory Response and Neuron Cell Apoptosis
Source: Int J Mol Sci. 2024 Sep 25;25(19):10321. doi: 10.3390/ijms251910321 (PMC11476780; doi:10.3390/ijms251910321)
Supplement: Supplementary file 1 [file ijms-25-10321-s001.zip › ijms-3122635-supplementary.pdf]

Supplementary Table S1. Antibody list

**First Antibody**	**Dilution Concentration**	**Brand**	**Product Number**
Cluster of Differentiation 206 (CD206)	1:1000	Proteintech, Illinois, USA	18704-1-AP
Cluster of Differentiation 68 (CD68)	1:500	abcam, Cambridge, UK	ab955
Arginase 1 (Arg1)	1:500	Proteintech, Illinois, USA	16001-1-AP
Inducible nitric oxide synthase (iNOS)	1:500	BD, Franklin Lakes, USA	610329
Phosphorylated-Protein kinase B (p-Akt)	1:1000	Cell Signaling, Massachusetts, USA	4060
Protein kinase B (Akt)	1:1000	Cell Signaling, Massachusetts, USA	9272
β-actin	1:20,000	Sigma, St. Louis, USA	A5441
NAD-dependent deacetylase sirtuin-1 (SirT1)<br>Sirtuin 1 (SirT1)	1:500	Cell Signaling, Massachusetts, USA	9475
Nuclear factor erythroid 2-related factor 2 (Nrf2)	1:500	Proteintech, Illinois, USA	16396-1-AP
Phosphorylated-nuclear factor kappa-light-chain-enhancer of activated B cells (p-NF-kB)	1:1000	Cell Signaling, Massachusetts, USA	3033
NF-kB	1:1000	Cell Signaling, Massachusetts, USA	6956
Phosphorylated-nuclear factor of kappa light polypeptide gene enhancer in B-cells inhibitor α (p-IkB-α)	1:1000	Cell Signaling, Massachusetts, USA	2697
B-cell leukemia/lymphoma 2 protein (Bcl-2)	1:500	Proteintech, Illinois, USA	12789-1-AP
Bcl2-associated X protein (Bax)	1:2000	Proteintech, Illinois, USA	50599-2-1g
Cleaved Caspase-3	1:200	Cell signaling, Massachusetts, USA	9664
Caspase-3	1:1000	Cell Signaling, Massachusetts, USA	9662
N-methyl-D-aspartate receptor 2A (NMDAR2A)	1:500	Sigma-Aldrich, St. Louis, USA	AB1555P
N-methyl-D-aspartate receptor 2B (NMDAR2B)	1:500	Sigma-Aldrich, St. Louis, USA	AB1557P
Brain derived neurotrophic factor (BDNF)	1:500	ABGENT, California, USA	AG1181
Tropomyosin receptor kinase B	1:500	ABGENT, San Diego, USA	AN1211

(TrkB)			
Phosphorylated-extracellular signal-regulated kinase (p-ERK)	1:1000	Cell Signaling, Massachusetts, USA	9101
Extracellular signal-regulated kinase (ERK)	1:1000	Cell Signaling, Massachusetts, USA	9102
Phosphorylated-cAMP-response element binding protein (p-CREB)	1:500	Cell Signaling, Massachusetts, USA	9198

**Secondary Antibody**	**Dilution Concentration**	**Brand**	**Product Number**
Peroxidase AffiniPure™ Goat Anti-Rabbit IgG (H+L)	1:2000	Jackson ImmunoResearch, Pennsylvania, USA	111-035-144
Peroxidase AffiniPure™ Goat Anti-Mouse IgG (H+L)	1:2000	Jackson ImmunoResearch, Pennsylvania, USA	115-035-146
